# Chronic Chrysin, but Not Diazepam, Induces Anxiolytic-like Effects Without Behavioral Tolerance

**DOI:** 10.3390/molecules31183177

**Published:** 2026-09-10

**Authors:** Luz María Nicio-Antonio, Gabriel Guillén-Ruiz, Ana Karen Limón-Vázquez, Jonathan Cueto-Escobedo, León Jesús Germán-Ponciano, Emma Virginia Herrera-Huerta, David Armando Argüello-Velasco, Juan Francisco Rodríguez-Landa

**Affiliations:** 1 Posgrado en Neuroetología, Instituto de Neuroetología, Universidad Veracruzana, Xalapa-Enriquez 91190, Veracruz, Mexico; zs23024853@estudiantes.uv.mx (L.M.N.-A.); zs24025417@estudiantes.uv.mx (D.A.A.-V.); 2 Laboratorio de Neurofarmacología, Instituto de Neuroetología, Universidad Veracruzana, Xalapa-Enriquez 91190, Veracruz, Mexico; analimon@uv.mx (A.K.L.-V.); lgerman@uv.mx (L.J.G.-P.); 3 Programa de Investigadoras e Investigadores por México, SECIHTI-Instituto de Neuroetología, Universidad Veracruzana, Xalapa-Enríquez 91190, Veracruz, Mexico; 4 Departamento de Investigación Clínica y Traslacional, Instituto de Ciencias de la Salud, Universidad Veracruzana, Xalapa-Enriquez 91190, Veracruz, Mexico; jcueto@uv.mx; 5 Facultad de Ciencias Químicas, Universidad Veracruzana, Orizaba 94340, Veracruz, Mexico; emherrera@uv.mx

**Keywords:** anxiolytic, chrysin, GABA_A_ receptor, pharmacological tolerance

## Abstract

Background: Chrysin (5-7-dihydroxyflavone) exerts anxiolytic-like effects via the GABA_A_/benzodiazepine receptor complex after acute administration, similar to diazepam. Chronic diazepam treatment produces pharmacological tolerance, likely due to structural receptor modification. Whether chronic chrysin treatment also produces pharmacological tolerance remains unknown. This study evaluated GABA_A_/benzodiazepine receptor-mediated behavioral responses after a pharmacological challenge with a sedative dose of diazepam (5 mg/kg) in rats chronically treated with chrysin or diazepam. Methods: Thirty-five male Wistar rats were divided into five groups: vehicle, chrysin (1, 2.5, or 5 mg/kg), and diazepam (2 mg/kg, pharmacological control). Treatments were injected intraperitoneally for 32 days. On day 28, rats were evaluated in the elevated plus maze and locomotor activity tests. On day 32, all groups received a pharmacological challenge with 5 mg/kg diazepam and were assessed via sedative scale and the rota-rod test. Results: Chronic Chrysin (5 mg/kg) but not diazepam, maintained anxiolytic-like effects. No doses of chrysin showed pharmacological cross-tolerance to 5 mg/kg of diazepam, which produced incoordination and sedation, similar to vehicle. Conclusions: Chronic treatment with chrysin (5 mg/kg) maintained their anxiolytic-like effects without pharmacological tolerance or cross-tolerance, unlike diazepam. This result supports the potential anxiolytic-like effects of chrysin in the long-term, as an alternative for anxiety disorders that require chronic treatment. This could contribute to the translational studies that could be applied to humans in the future.

## 1. Introduction

Anxiety disorders include cognitive, emotional, and physiological symptoms [1]. Their etiology has been associated with dysfunctions in some neurotransmitter systems [2], particularly the GABAergic system regulated by GABA_A_ receptors. This ionotropic receptor has several binding sites to recognize different drugs, such as agonists or allosteric modulators, producing anxiolytic, sedative, and hypnotic effects, among others [3].

The first-line medications used to treat these disorders are selective serotonin reuptake inhibitors (SSRIs) such as fluoxetine, which are effective in reducing anxiety symptoms; however, their therapeutic effects do not become apparent until two to three weeks after treatment begins, while adverse effects are often severe during the first few weeks [4]. The primary mechanism of action of SSRIs involves presynaptic 5-HT1A receptors, facilitating serotonergic actions on postsynaptic 5-HT2A receptors [5].

Other medications used to treat anxiety are those that act on the GABAergic system, including benzodiazepines. However, they are used only in specific anxiety conditions when the patient’s life is at risk; their use is not recommended for prolonged periods because they can lead to physical and psychological dependence, as well as benzodiazepine tolerance and a loss of therapeutic effects [6]. This effect has been related to possible alterations in the configuration of the α1 and α5 subunits of the GABA_A_ receptor, which is necessary for their therapeutic effects [7].

In addition, some patients seek alternative therapies to treat anxiety, such as certain plant-based substances containing active ingredients that act on the central nervous system. Unfortunately, in most cases, the neurobiological mechanisms of these substances have yet to be identified, preventing their safe use in the treatment of anxiety in humans.

Studies in zebrafish [8], mice [9,10] and rats [11,12] indicate that some phytochemicals, including flavonoids like chrysin, exhibit anxiolytic- and antidepressant-like effects. The flavonoid chrysin (5,7-dihydroxyflavone), present in *Passiflora coerulea*, propolis and honey [10], exerts anxiolytic-like effects in male and ovariectomized female rats following acute treatment [13] with the participation of the GABA_A_ receptor [11,12]. The anxiolytic-like effect of chrysin likely is produced by its interaction with the α5 subunit of the GABA_A_ receptor, as suggested in previous studies [8]. Consistently, in vitro studies conducted on *Xenopus laevis* oocytes have shown that chrysin can act directly on the α1, β1, and γ2 subunits of the GABA_A_ receptor [14], subunits associated with the mechanism of action of drugs with anxiolytic properties, but the effect of chrysin during long-term treatment, as well as its potential effects on benzodiazepine tolerance through GABA_A_ receptor, has not been investigated in rodents.

Despite the similarities in the anxiolytic actions produced by chrysin and diazepam, the mechanism of action of chrysin may differ from that of benzodiazepines. In acute treatments, chrysin does not produce motor hypoactivity, an effect commonly associated with benzodiazepine treatment [13,15,16,17,18], indicating that other neuronal and neurochemical mechanisms could be involved. In fact, the anxiolytic and antidepressant-like actions of chrysin are similar to those produced by the administration of progesterone and allopregnanolone [17].

Altogether, these findings suggest that chrysin could be an alternative to benzodiazepines for the treatment of anxiety disorders; nonetheless, the effect of chronic chrysin treatment on anxiety-like behavior and its potential to produce pharmacological tolerance involving the GABA_A_ receptor requires further exploration.

This study evaluated the anxiolytic-like effect of chronic treatment with chrysin and compared it with the effects produced by diazepam. Additionally, the pharmacological response of all treatments to a sedative dose of 5 mg/kg diazepam was explored. This pharmacological challenge was utilized to assess whether the flavonoid chrysin could induce long-term pharmacological cross-tolerance to benzodiazepines, through the GABA_A_ receptor, comparable to that produced by diazepam [18].

## 2. Results

### 2.1. Chronic Treatment with Chrysin or Diazepam on Anxiety-like Behavior

#### 2.1.1. Elevated Plus Maze

**Figure 1:**
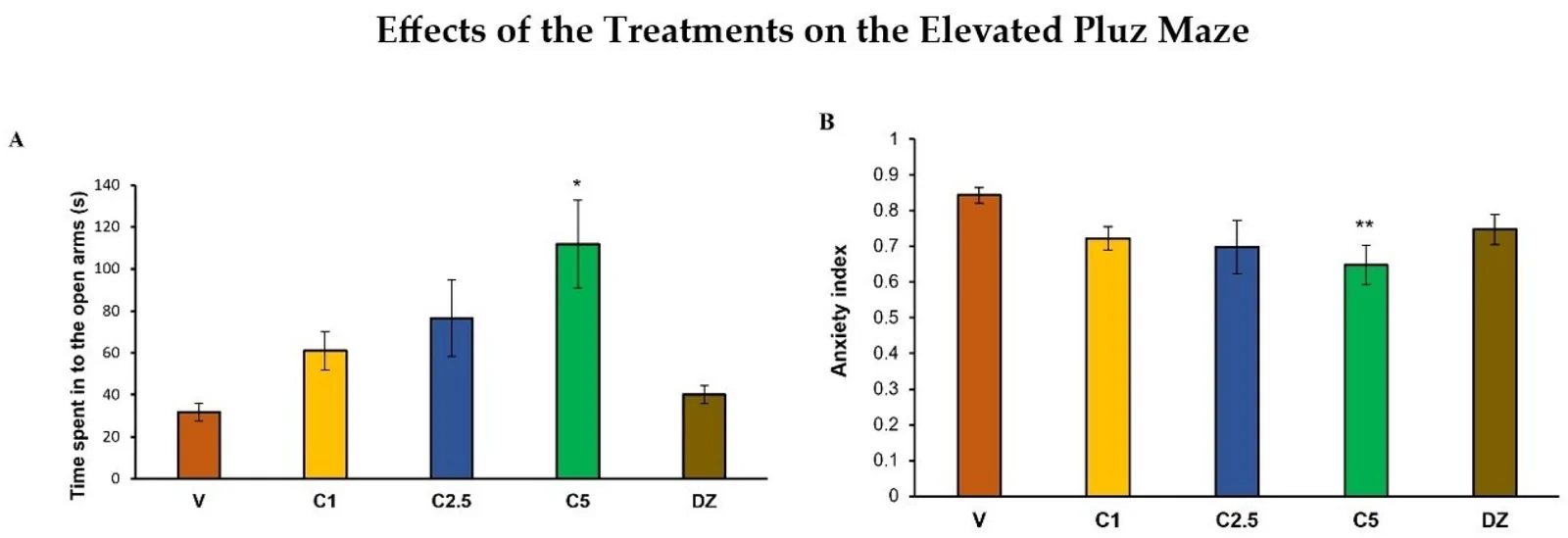
Elevated plus maze tests. (**A**) Time spent in open arms and (**B**) anxiety index, * *p* < 0.05 vs. V and DZ, ** *p* < 0.05 vs. V. Dunn’s post hoc test. V = vehicle, C1 = chrysin 1 mg/kg, C2.5 = chrysin 2.5 mg/kg, C5 = chrysin 5 mg/kg, DZ = diazepam 2 mg/kg. All treatments were intraperitoneally injected. The tests were conducted on day 28 of treatment, one hour after the last injection.

The statistical analysis revealed a significant difference in time spent in open arms [H_(4)_ = 19.377, *p* < 0.001] according to the treatments. The post hoc test indicated that the group treated with 5 mg/kg chrysin spent more time in open arms compared to the vehicle and 2 mg/kg diazepam groups. The highest dose of 5 mg/kg chrysin produced the greatest effect on the open arms. Chronic treatment with 2 mg/kg diazepam showed similar values to those found in the vehicle group (Figure 1A).

The analysis of the anxiety index revealed significant effects between treatments [H_(4)_ = 9.698, *p* = 0.046]. The post hoc test showed that 5 mg/kg chrysin produced lower anxiety-like behavior than the vehicle group (Figure 1B).

#### 2.1.2. Locomotor Activity Test

The analysis showed no significant effect of the treatment on the number of crossings [F_(4,30)_ = 2.318, *p* = 0.080]; however, changes were observed in the time spent on grooming [F_(4,30)_ = 3.328, *p* < 0.023] and rearing [F_(4,30)_ = 3.546, *p* < 0.017]. The post hoc test revealed that 1 mg/kg chrysin increased grooming behavior compared to the vehicle group. Additionally, 2.5 mg/kg chrysin increased the time spent on rearing compared to diazepam, but no differences were detected versus the vehicle group (Table 1).

#### 2.1.3. Sedation Scale

**Figure 2:**
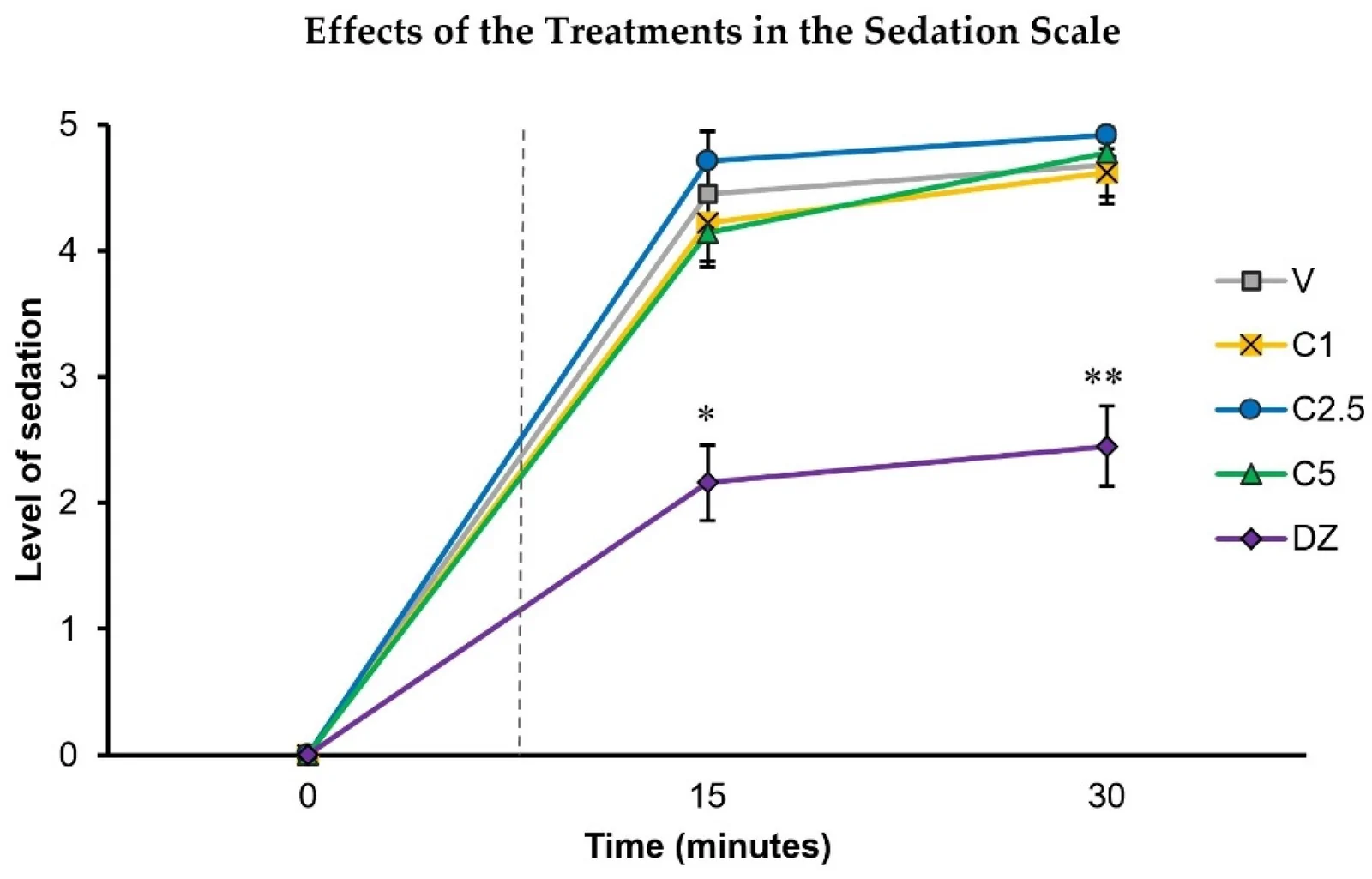
Sedation scale. Effects of the treatments on sedation level. * *p* < 0.001 vs. all groups at 15 and 30 min; ** *p* < 0.001 vs. V, C2.5 at 15 and 30 min, and C1, C5 at 30 min. V = vehicle, C1 = chrysin 1 mg/kg, C2.5 = chrysin 2.5 mg/kg, C5 = chrysin 5 mg/kg, DZ = diazepam 2 mg/kg. Bonferroni post hoc test. Time 0 is shown representatively as the start of the evaluation (before the dotted line); however, it was not included in the statistical analysis due to restriction of the statistical test. All treatments were intraperitoneally injected. The tests were conducted on day 28 of treatment one hour after the last injection.

The analysis of the sedation level revealed significant differences according to the treatments [χ^2^_(4)_ = 17.96, *p* = 0.001] and observation time [χ^2^_(1)_ = 4.89, *p* = 0.027], but not for the interaction between the factors [χ^2^_(4)_ = 1.01, *p* = 0.909]. The results showed a sedation stage from 15 min through 30 min post-injection, which was the observation cut-off point in this study. The diazepam group exhibited the lowest level of sedation from 15 min and continued through 30 min post-administration. In contrast, the vehicle and the chrysin-treated groups showed higher sedation levels from 15 min until the end of the evaluation (Figure 2).

#### 2.1.4. Rota-Rod Test

**Figure 3:**
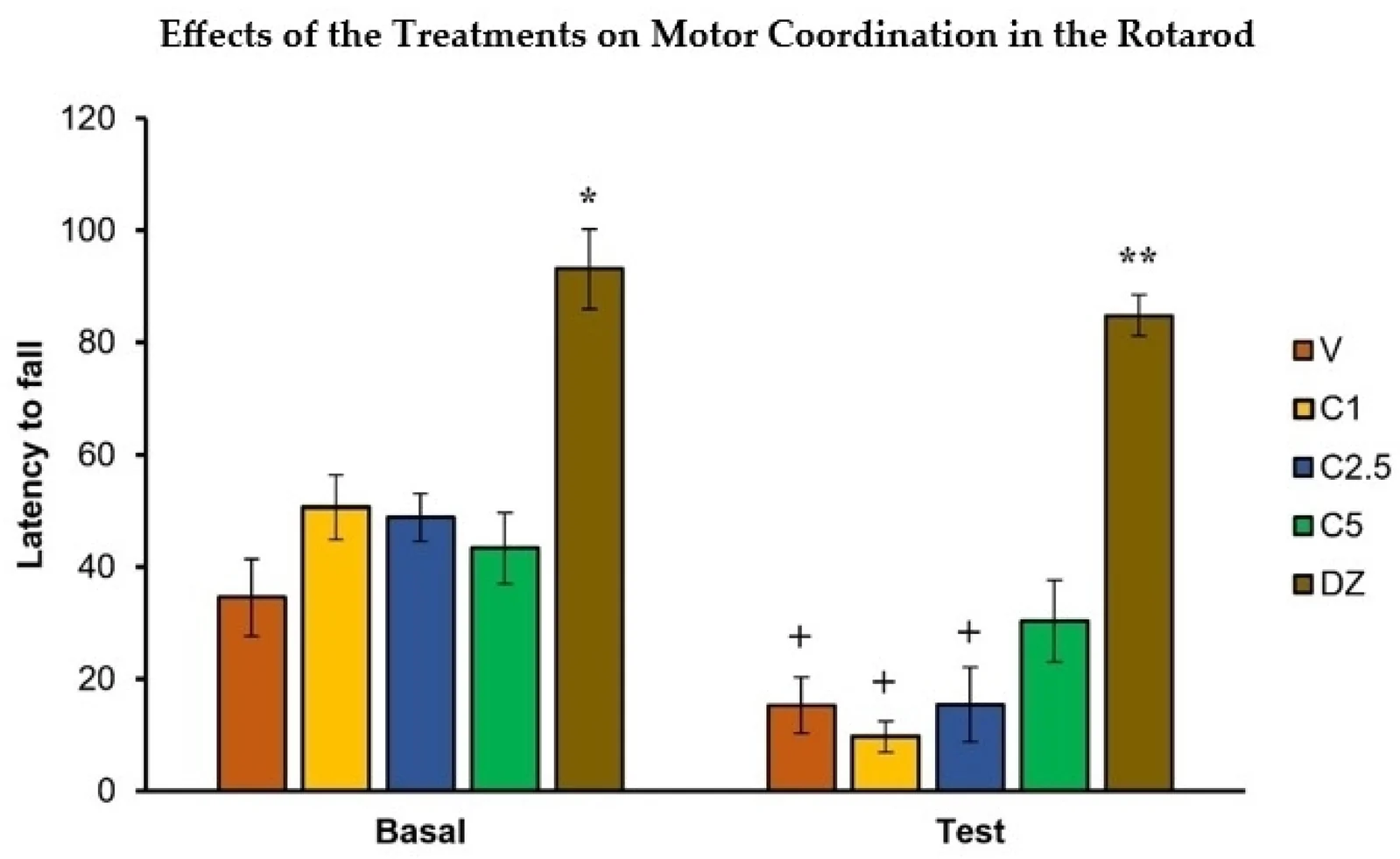
Rota-rod test. Effect of treatment on latency to fall. * *p* < 0.05 vs. all groups in baseline; ** *p* < 0.05 vs. all groups in test session; + *p* < 0.05 vs. same group baseline. V = vehicle, C1 = chrysin 1 mg/kg, C2.5 = chrysin 2.5 mg/kg, C5 = chrysin 5 mg/kg, DZ = diazepam 2 mg/kg. Student–Newman–Keuls post hoc test. All treatments were intraperitoneally injected. The tests were conducted on day 28 one hour after the last injection.

The analysis indicated significant differences between treatments [F_(4,30)_ = 38.216, *p* < 0.001], observation time [F_(4,30)_ = 43.665, *p* < 0.001], and the interaction between factors [F_(4,30)_ = 3.021, *p* < 0.033]. The post hoc test revealed that animals treated with 2 mg/kg diazepam had a longer latency to fall compared to the vehicle and chrysin treatment. The chrysin-treated groups exhibited a shorter latency to fall, similar to the vehicle group. The 2 mg/kg diazepam group had a longer latency compared to the vehicle group, both in the baseline session and during the pharmacological challenge with the sedative dose of 5 mg/kg diazepam. In the baseline session, the groups treated with vehicle and 1, 2.5 and 5 mg/kg chrysin had latency times similar to the vehicle group. However, in the test session, this variable was significantly reduced in the vehicle and 1 and 2.5 mg/kg chrysin groups after the pharmacological challenge with 5 mg/kg diazepam. The 5 mg/kg chrysin group attenuated the effect of the sedative dose of diazepam in the pharmacological challenge on this variable (Figure 3).

## 3. Discussion

This study evaluated the anxiolytic-like effect of chronic treatment with different doses of the flavonoid chrysin in comparison with diazepam, and the potential development of pharmacological tolerance to benzodiazepines by chrysin involving the participation of the GABA_A_/benzodiazepine receptor. The main results are: (a) chronic treatment with 5 mg/kg chrysin, but not 2 mg/kg diazepam, exerted anxiolytic-like effects; (b) none of the treatments produced motor alterations in the locomotor activity test; and (c) the pharmacological challenge with 5 mg/kg diazepam in all groups produced sedation and motor incoordination in the chrysin-treated groups, while the diazepam-treated group was devoid of these effects. The results suggest that long-term treatment with chrysin did not produce pharmacological cross-tolerance to diazepam effects, while long-term treatment with diazepam produced tolerance, possibly through adaptations of the GABA_A_/benzodiazepine receptor. In consequence, this study supports the possibility that chrysin would be an alternative for the treatment of anxiety disorders that requires long-term treatments, opening new avenues of research to support translational studies that can then be evaluated in humans.

It is necessary to distinguish between three distinct phenomena that are related but not equivalent. First, pharmacological tolerance to chrysin itself refers to the loss of the anxiolytic-like effect following chronic administration; this phenomenon was analyzed directly by evaluating animals in the elevated plus-maze test after 28 days of administration, a period during which 5 mg/kg chrysin maintained its anxiolytic effect, suggesting an absence of behavioral tolerance to the anxiolytic action of this flavonoid. Second, cross-tolerance with diazepam concerns whether chronic exposure to chrysin alters the animals’ behavioral sensitivity to a subsequently administered, pharmacologically distinct ligand of the GABA_A_/benzodiazepine site (diazepam); this was evaluated indirectly via a pharmacological challenge using a sedation scale and the rota-rod test in response to an acute, sedative dose of diazepam (5 mg/kg), following the operational definition of cross-tolerance between GABAergic drugs sharing the same target receptor [19]. Groups receiving chronic chrysin treatment exhibited sedative responses and motor impairments in response to the sedative dose of diazepam that were similar to those of the vehicle group, suggesting an absence of behavioral cross-tolerance; in contrast, the group treated chronically with diazepam showed an attenuated response consistent with tolerance, specifically, a lower level of sedation and no impairment of coordination on the rota-rod. Third, adaptation at the receptor level refers to molecular changes in the GABA_A_ receptor, such as modifications in the configuration of the subunit, phosphorylation state, or the coupling between GABA and benzodiazepine binding sites, that underlie the development of pharmacological tolerance and cross-tolerance with benzodiazepines [18,20]. This study did not include molecular or electrophysiological measurements of the GABA_A_ receptor and, therefore, did not directly assess adaptation at the receptor level; however, the behavioral findings presented here could be consistent with the absence of such adaptation following chronic treatment with chrysin, unlike what occurs with diazepam, as reported in other studies [20,21].

The elevated plus maze is widely used to evaluate anxiolytic-like effects of different drugs such as benzodiazepines, e.g., diazepam and chlordiazepoxide [22,23,24], antidepressants with anxiolytic effects, such as fluoxetine [25,26], neurosteroids, e.g., 17β-estradiol [27,28], progesterone [29], allopregnanolone [30,31], as well as natural substances like the phytoestrogen genistein [32,33] and the flavonoid chrysin [31]. In this apparatus, exploration of the open arms is the main indicator of an anxiety state. In the present study, an anxiolytic-like effect of 5 mg/kg chrysin was observed after chronic administration, similar to that reported with acute administration [11,34], and that of positive modulators of the GABA_A_ receptor such as neurosteroids, barbiturates and benzodiazepines [35]. However, in this study, chronic administration of diazepam failed to exhibit an anxiolytic effect. In this regard, benzodiazepines like diazepam, allosterically modulate the actions of GABA, but chronic treatment has been associated with adverse effects that involve changes in the α-subunit composition of the GABA_A_ receptor. Tolerance to the anxiolytic effects of diazepam is associated with alterations in the density of receptors containing the α1 subunit, while tolerance to the sedative effects is associated with a decoupling between the GABA_A_ receptor and the benzodiazepine binding site, possibly due to increased phosphorylation of the γ2 subunits [18]. These changes lead to receptor desensitization, promoting tolerance and pharmacological resistance [36,37]. This produces the loss of its anxiolytic effect in humans and laboratory animals [38,39,40].

In contrast, chrysin did not show tolerance and maintained its anxiolytic-like effects after chronic administration, increasing the time spent in open arms and reducing the anxiety index.

Although it has been suggested that chrysin acts on subunits that recognize benzodiazepines [12], it does not seem to modify receptor structure in the long term, preserving its anxiolytic efficacy as has been documented with acute treatment. Acute administration of 2 and 4 mg/kg chrysin has been shown to have an anxiolytic-like effect similar to that of diazepam, but without the motor effects characteristic of diazepam, while pretreatment with picrotoxin, a GABA_A_ receptor antagonist, canceled the anxiolytic-like effects of the aforementioned doses, suggesting its activity in the GABAergic system [12,41]. In fact, the anxiolytic- and antidepressant-like actions of chrysin are similar to those produced by progesterone and allopregnanolone through the GABA_A_ receptors [15,17,34,42].

These effects have also been observed in female zebrafish, in which, the acute effects of 1 mg/kg chrysin were blocked by pretreatment with picrotoxin, suggesting the involvement of the GABA_A_ receptor in anxiolytic-like effects. Furthermore, molecular docking results suggest that these effects may be associated with interactions involving the α5 subunit of the GABA_A_ receptor [8], suggesting that the effects of chrysin on anxiety-like behavior may be similar to those of diazepam, due to the subunit through which it exerts its effects, but without modifying the motor activity. The mechanism by which chrysin lacks tolerance remains unexplored.

Chronic diazepam treatment activates the α1 and α5 subunits of the GABA_A_ receptor, associated with tolerance to sedative effects [21,43]. Consistently, in this study, chronic diazepam did not show sedative effects in the sedation scale, possibly due to the changes in the receptor after chronic treatment, which also regulate motor action. Studies comparing the effects of 2, 4 and 8 µmol kg^−1^ chrysin and 7 µmol kg^−1^ diazepam in acute treatment in male Wistar rats have shown no motor changes related to the treatments that could interfere with behavioral variables such as grooming [34]. Grooming as self-directed behavior is an indicator of the motivational and emotional state of the rat [44]. In the present study, grooming time increased in the group treated with 1 mg/kg chrysin compared to the vehicle and diazepam groups, suggesting that grooming could be related to motivated behavior [34], while the lack of effect in the diazepam group is possibly due to a change at the level of the GABA_A_ receptor.

Rearing behavior is performed when the animal stands on its hind limbs to explore a novel environment and involves a certain level of motor coordination. In this study, we did not observe any alteration in rearing behavior with the administered treatments, suggesting that the treatments do not modify the motor component associated with exploratory behavior. Subsequently, the locomotor activity test confirmed the absence of motor changes that could interfere with our results.

Therefore, the effects observed in the elevated plus maze can be attributed to anxiety rather than non-specific motor components. Considering that the flavonoid chrysin exerts effects similar to diazepam in behavioral tests, we evaluated whether administration of a sedative dose of diazepam could trigger adaptations leading to tolerance similar to that observed with benzodiazepines. Chronic treatment with 2 mg/kg diazepam produces hypoactivity in patients [32], while in animal models, a single dose of 5 mg/kg causes motor incoordination [45]. Sedation was evaluated using a scale where the level of sedation of the animals is classified into 5 levels, where 0 indicates absence of sedation, while 4 represents deep sedation and complete inactivity [45]. This scale has also been used to evaluate the sedative effects of 5 mg/kg diazepam [45,46].

In this study, we observed that groups receiving chronic treatment with chrysin (1, 2.5 and 5 mg/kg) had higher levels of sedation during the pharmacological challenge with 5 mg/kg diazepam, while the chronic diazepam-treated group showed lower levels of sedation. This suggests that chronic diazepam dampened the response of the organism to a higher dose, possibly by inducing changes at the GABA_A_ receptor, since the expected sedative effects were not detected, unlike in the vehicle and chrysin-treated groups. Subjects with an unstimulated receptor would be expected to show a higher level of sedation, while changes induced by chronic treatment would lead to decreased sedation [47], as observed in the present study.

Studies conducted in male Wistar rats treated with 2 mg/kg diazepam for 28 days showed the development of dependence, generating anxiety-like behaviors observed in the elevated plus maze test [21]. In this sense, the participation of GABA_A_ receptors containing the α1 subunit is related to pharmacological dependence [48]. Consistently, studies in male rats treated with 15 mg/kg diazepam for 14 days showed tolerance to the anxiolytic actions in the elevated plus maze, associated with an increase in the percentage of GABA_A_ receptors with the α1 subunit in the cerebral cortex and increased phosphorylation levels of the γ2 subunit from day 7, persisting until day 14 of treatment [18]. The concomitant activation of the α1 and α5 subunits contributes to the development of tolerance to the sedative actions of diazepam [21]. This coincides with our observations, where chronic diazepam treatment produced the loss of the effect of the sedative dose, while chrysin did not produce this loss of effect. Also, it has been observed that long-term chrysin has behavioral effects similar to neurosteroids and fluoxetine [49]. In addition, 5 mg/kg chrysin has shown antidepressant-like effects similar to drugs with serotonergic activity [15]. In this study, it is not possible to discard the involvement of other neurotransmission systems that regulate the anxiolytic effect without generating long-term tolerance.

The anxiolytic effects of chrysin, similar to those of diazepam, are due principally to its actions on the GABA_A_ receptor, without excluding other neurotransmission systems like the serotonergic and dopaminergic systems, which also are modified by chronic treatment with chrysin [9,15,50]. Particularly, the effects of chrysin on the GABA_A_ receptor are canceled by the prior administration of flumazenil, an antagonist of the benzodiazepine binding site of the GABA_A_ receptor [11]. Also, pretreatment with picrotoxin, an antagonist of the GABA_A_ receptor chloride channel, cancels out the anxiolytic-like effects of chrysin and allopregnanolone, suggesting that chrysin activates the GABAergic system, activating other binding sites in addition to the benzodiazepine site, as occurs with the neurosteroid allopregnanolone [13,17,51]. Evidence indicates that anxiolytic-like effects of chrysin are not only established in the GABAergic system but also in other systems such as the serotonergic system [15].

In this regard, a study conducted on male Wistar rats for 28 days evaluated the effect of low (1 mg/kg) and high (5 mg/kg) doses of chrysin, compared with allopregnanolone, a neurosteroid with GABAergic activity, and fluoxetine, a drug with serotonergic activity. The data showed that low-dose chrysin has an effect similar to the neurosteroid, while the high dose maintains an effect similar to that of fluoxetine in the total immobility time in the forced swim test. The effect of chrysin at low doses was canceled by picrotoxin but not the effects produced at high doses [47], implying that the possible mechanism of action of chrysin does not involve only one system.

This would explain in some way why the effects of chrysin after prolonged treatment do not lose their anxiolytic effect, as occurs with diazepam.

The rota-rod test is used to detect alterations in motor coordination caused by pharmacological treatments, particularly the sedative effects of CNS-active substances. Acute administration of a sedative dose of 5 mg/kg diazepam reduces the latency to fall, which is related to muscular relaxation and motor incoordination [50]. A healthy animal will have a longer latency to fall, as it will remain on the rotating roller longer, maintaining coordination and balance, which are interpreted in this test as a baseline state, while in an animal treated with sedative drugs, the latency to fall will be shorter, as there is reduced balance and motor coordination [52]. In this study, it was observed that the pharmacological challenge with diazepam exerted sedative effects in animals chronically treated with chrysin, similar to the vehicle. Meanwhile, the diazepam group showed normal motor coordination and remained on the rota-rod for a longer time, suggesting that chronic diazepam promotes the development of pharmacological tolerance at the GABA_A_/benzodiazepine receptor, as previously reported [18,48], but this tolerance did not occur with 1 and 2.5 mg/kg chrysin. Notably, 5 mg/kg chrysin did not exhibit differences in latency to fall between baseline and test sessions compared to the vehicle group.

It is worth noting that the diazepam group exhibited a longer latency to fall than the other groups, starting from the baseline session. Given that diazepam and its active metabolite, desmethyldiazepam, are rapidly eliminated in rats (plasma and brain half-lives of approximately 1 h following an intraperitoneal dose) [53], the presence of residual drug at the time of testing (24 h after the last injection) is an unlikely explanation. A more plausible explanation, albeit one not directly evaluated here, involves a potential neuroadaptive change in the GABA_A_ receptor resulting from repeated diazepam exposure; indeed, reports indicate that chronic daily intraperitoneal administration of 0.06 mg/kg diazepam for five days in adult male mice induces sensitization of motor activity, manifested as increased distance traveled, an effect possibly linked to the activation of the α1 subunit of the GABA_A_ receptor [54]. Although the cited study assessed distance traveled rather than motor coordination, it demonstrates that chronic diazepam treatment alters GABA_A_ receptor subunit conformation, thereby affecting the motor function of the subjects, a category that encompasses the motor coordination assessed via the rota-rod test. However, as our study did not analyze latency to fall data from the training sessions, this represents a limitation that should be addressed in future research to determine whether the difference in baseline values between the diazepam group and the other groups developed progressively over the three days of training and whether it was associated with changes in GABA_A_ receptor subunits.

Also, there is the possibility that this dose of chrysin impacts other neurotransmission systems, given reports that indicate that this dose modifies the expression of 5-Hydroxytryptamine 1A (5HT-_1A_) and 5-Hydroxytryptamine 2A (5HT-_2A_) receptors [15], as well as serotonin levels in the brain [9]. Thus, the evidence indicates that chrysin mechanisms are regulated by both GABAergic and serotonergic systems. In the present study, chronic administration of 5 mg/kg chrysin showed no significant differences between baseline and test sessions in the rota-rod test. Although this study indirectly evaluated the pharmacological response of the GABA_A_ receptor, future studies should explore the involvement of other receptors implicated in the etiology of anxiety, such as the serotonergic system, to better understand the mechanisms underlying the long-term anxiolytic effects of chrysin.

Unfortunately, the present study did not directly measure changes in the density of the GABA_A_ receptor, which constitutes a significant limitation regarding the use of a diazepam pharmacological challenge; likewise, it is plausible that the 5 mg/kg dose of chrysin exerts its anxiolytic effect via other systems while simultaneously inducing changes in GABAergic receptors, thereby attenuating the sedative effect of diazepam during the challenge, a hypothesis that cannot be confirmed using the diazepam pharmacological challenge. Nevertheless, there is extensive literature suggesting that pharmacological tolerance to benzodiazepines, particularly diazepam, is linked to these changes in the subunits of the GABA_A_ receptor. Tolerance to anticonvulsant effects of diazepam has been associated with a decrease (37%) in the expression of the α1 subunit in the frontoparietal motor cortex, and an increase (221%) in α5 subunits was measured in all cortical layers; whereas imidazenil, a partial allosteric modulator of GABA, does not produce tolerance nor significant changes in the expression of any of the GABA_A_ receptor subunits studied [55]. Similarly, a three-week treatment using subcutaneous silastic reservoirs containing 90 mg of crystalline diazepam decreased the α1 subunit mRNA levels in the hippocampus by 40%, though not in the cortex or cerebellum. The α5 subunit mRNA level decreased in the cerebral cortex (28%) and hippocampus (15%), and γ2 subunit mRNA levels decreased (40%) in the cortex. Diazepam treatment did not affect mRNA levels for the α2, α3, α4, β2, or β3 subunits [56]. Similar effects have been observed with flurazepam, suggesting that this mechanism underlies benzodiazepine tolerance [57]. This evidence justifies the use of the diazepam pharmacological challenge in this first research; however, future research should also explore other mechanisms, such as GABA_A_ receptor trafficking, the effects of intracellular changes mediated by transcriptional and neurotrophic factors, ionotropic glutamate receptors, other neurotransmitters (serotonin, dopamine, and acetylcholine), and neurosteroids [21,58].

An additional consideration is the pharmacokinetic profile of chrysin. In humans, orally administered chrysin exhibits very low systemic bioavailability (estimated at <1%), primarily due to extensive intestinal and hepatic phase II metabolism (glucuronidation and sulfation reactions) and the activity of efflux transporters, with the majority of the unabsorbed dose recovered in the feces [58]. In the present study, chrysin was administered intraperitoneally, a route that bypasses intestinal first-pass metabolism and can thus achieve greater systemic exposure compared to equivalent oral doses; this aligns with the behavioral effects observed in this study, as well as in previous studies by our group [13,17,35,49] and others [9,11,12,16] using the same route. However, there are no studies determining the pharmacokinetic parameters (plasma or brain concentrations, and half-life) of chrysin at the intraperitoneal doses used (1–5 mg/kg); this limits direct comparison with reported oral bioavailability data in humans and highlights an area for future research, particularly if chrysin is to be considered for oral therapeutic use.

It should also be clarified that this study used only male rats as an initial approach to determine whether chronic treatment with chrysin could induce pharmacological tolerance at the GABA_A_ receptor, assessed indirectly through behavioral tests and a pharmacological challenge. Female rats were not included in this initial experiment because fluctuations in ovarian hormones across the estrous cycle are known to influence anxiety-related behaviors by modulating the GABA_A_ receptor, particularly hormones like progesterone and their reduced metabolite allopregnanolone. Elevated hormone levels during proestrus-estrus are associated with anxiolytic-like effects, whereas their decline during metestrus-diestrus is associated with anxiogenic-like effects [59,60]. Consequently, including females in this initial study would have required controlling for the estrous cycle phase, thereby increasing the number of animals required, which was not justified from a bioethical standpoint (the 3Rs principle) for this initial exploratory design. Using males made it possible to avoid the significant influence of steroid hormone fluctuations while simultaneously reducing the number of animals used. The importance of including sex as a variable in studies is acknowledged; therefore, future research should address the chronic effects of chrysin and its relationship to pharmacological tolerance in female rats, considering the influence of the estrous cycle phase.

## 4. Materials and Methods

### 4.1. Ethics

The experimental procedures followed international (Guide for the Care and Use of Laboratory Animals; The National Institute of Health, 1996) [61] and national (Especificaciones Técnicas para la Producción, Cuidado y Uso de Animales de Laboratorio; NOM-062-ZOO-1999) [62] ethics guidelines. Additionally, the 3Rs (reduce, replace, refine) were applied to minimize animal discomfort [63]. The general protocol was approved by Comité Interno para el Cuidado y Uso de Animales de Laboratorio del Instituto de Ciencias de la Salud, Universidad Veracruzana, Xalapa, México. Authorization: CICUAL-ICS_2024_03_002.

### 4.2. Animals

Thirty-five male Wistar rats, 2.5 months old, weighing 200–250 g at the beginning of the experiments, were included in the study. They were housed in transparent acrylic boxes (44 cm width × 33 cm length × 20 cm height), with 4 rats per box, under a 12 h/12 h light/dark cycle (lights on at 7:00 a.m.). The temperature was 25 °C ± 1 °C, and free access to purified water and food (Purina Pellets, Agribrands Purina México, Mexico City, Mexico) was supplied. The rats were habituated to the animal facility conditions for 4 weeks before starting the experimental manipulations.

### 4.3. Drugs

Dimethylsulfoxide (DMSO) was obtained from Golden Bell Reactivos (Mexico City, Mexico). Chrysin (5,7-dihidroxiflavone, >98% purity) was obtained from SIGMA-Aldrich Co. (ST. Louis, MO, USA). Diazepam (Valium 10^®^) was obtained from Hoffman-Roche Laboratory (Basel, Switzerland). Saline solution (0.9%) was purchased from PiSA Farmacéutica (Guadalajara, Jalisco, Mexico).

### 4.4. Experimental Procedures

Thirty-five rats were included in five independent groups (n = 7/group), one group received the vehicle (5% dimethylsulfoxide (DMSO) in physiological saline solution), this vehicle was selected based on previous studies by our group showing that, at this concentration, DMSO does not alter the behaviors of interest [13,49] furthermore, it falls midway between studies using the same vehicle at concentrations ranging from 0.05% [12] to 40% [11,16] without affecting behavioral assessments. Three groups received 1, 2.5, or 5 mg/kg chrysin, respectively (C1, C2.5, and C5, respectively), and the last group received 2 mg/kg diazepam. All treatments were injected intraperitoneally every 24 h for 28 consecutive days. The 2 mg/kg diazepam dose was used as the reference anxiolytic drug due to its GABAergic activity [49]. Chrysin doses were selected from previous studies showing its anxiolytic and antidepressant effects [9]. On day 28 of treatment, one hour after the last injection, each rat was evaluated in the elevated plus maze (5 min) and, subsequently, in the locomotor activity test (5 min).

**Figure 4:**
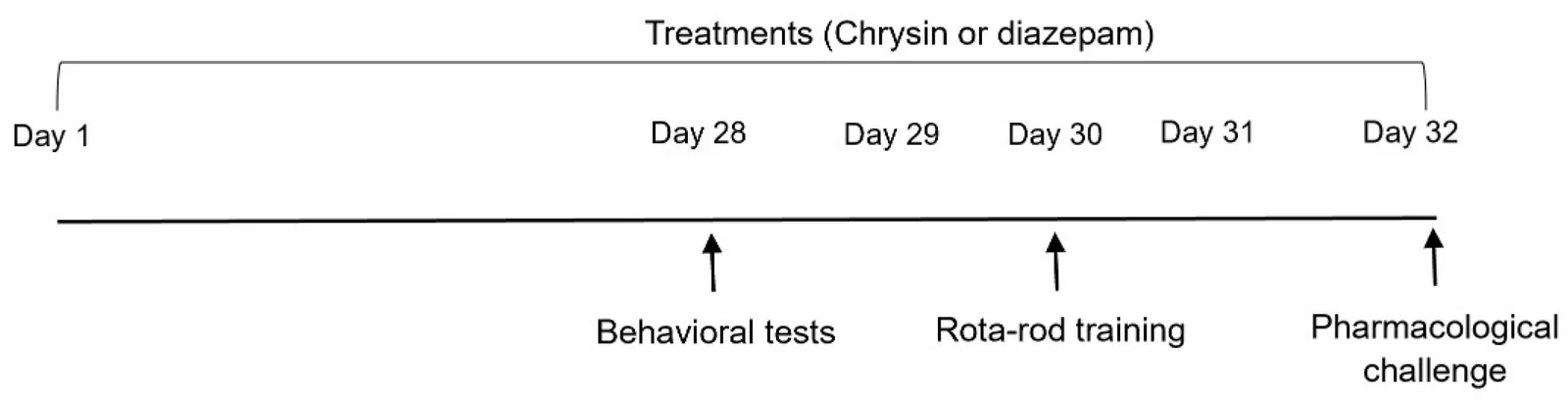
Timeline of the experiment. Sequence of pharmacological administrations and behavioral assessments.

The treatments were continued for 3 additional days, and training on the rota-rod test was initiated from day 29 to day 31 before the injection of treatments on these days. On day 31 of treatment (third day of training), a rota-rod assessment was conducted to establish the baseline. On day 32 of treatment, a pharmacological challenge was conducted by administering a sedative dose of 5 mg/kg diazepam 60 min after the last injection of the treatments. Immediately after the administration of the sedative doses (pharmacological challenge), the rats were individually observed for 30 min and scored according to the sedation scale at 15-, and 30- min post-injection [46] by an expert evaluator blinded to the treatments. Subsequently, the rats underwent the rota-rod test to evaluate their level of motor coordination. The sedative dose of diazepam (5 mg/kg) was selected from previous dose–response studies [64], which indicated moderate sedative effects beginning at 5 min and persisting up to 60 min. Figure 4 summarizes the timeline of the experiment.

The number of rats per group was determined using the statistical program G*Power V3.1.9.7 to calculate the sample size, considering an effect size of 0.8, α: 0.05, and power: 0.95. This was also based on previous studies by the research group, which evaluated the anxiolytic- and antidepressant-like effects of chrysin, as well as the involvement of the GABA_A_ receptor in these effects [13,17]. These studies have shown that 7 to 8 rats per group are sufficient to detect the pharmacological effects of chrysin without affecting the statistical power of the analysis. While a large sample size of animals per group may improve statistical power and reduce variability, according to the 3Rs in experimental research and restrictions imposed by the ethics committee, only 7 rats per group were authorized.

### 4.5. Behavioral Tests

The rats were individually evaluated in the elevated plus maze (5 min), followed by the locomotor activity test (5 min) to detect the effects of treatments on anxiety-like behavior and identify or rule out any motor effect that could interfere with the interpretation of variables in the elevated plus maze. In the pharmacological challenge, the rats received the sedative dose of diazepam and were evaluated using the sedation scale (at 15 and 30 min) and the rota-rod test (5 min) to determine the effect of the treatments.

#### 4.5.1. Elevated Plus Maze

The apparatus constructed from wood, featured two open arms and two closed arms arranged in a cross configuration. It was situated in a room illuminated at 40 lux. The dimensions of both the open and closed arms were 50 cm length × 10 cm width, with the closed arms further equipped with walls of 50 cm in height. The entire maze was elevated 50 cm above the floor. To evaluate the effects of the treatments, individual rats were placed in the center of the maze facing an open arm. The following variables were evaluated: (a) the time spent in the open arms and (b) the index of anxiety. These variables were selected because they are the most important for detecting the anxiolytic-like effect in this test [65]. Approximately 2 min after completing the elevated plus maze test, the rats were evaluated in the locomotor activity test. The elevated plus maze was cleaned with a 15% ethanol solution following each session.

#### 4.5.2. Locomotor Activity Test

The apparatus consisted of an opaque Plexiglas cage measuring 44 cm × 33 cm at the base, with walls 20 cm high. The floor was divided into 12 squares, each 11 cm × 11 cm. The test lasted 5 min, during which the following variables were evaluated individually: (a) the number of squares crossed (when the rat moved its hind legs from one square to another), (b) the time (seconds) of rearing (vertical posture of the rat with respect to the floor of the box), (c) the time (seconds) of grooming (when the rat performed circular movements in the cephalocaudal direction over the nose, ears, body, genital region, and tail). After each session, the locomotor activity test apparatus was cleaned with a 15% ethanol solution.

#### 4.5.3. Sedation Scale

To evaluate the state of sedation in animals chronically treated with chrysin or diazepam, a pharmacological challenge with diazepam was conducted. All groups that were injected with the different treatments for 28 consecutive days received, at the end, an intraperitoneal injection of 5 mg/kg diazepam (sedative dose). A trained observer blinded to the treatments evaluated the sedation state at 15 min and 30 min post-administration. The sedation scoring system ranged from 0 to 4, where 0 indicated alertness and full responsiveness, 1 signified alertness but reduced activity and ‘wobbly’ (instability), 2 represented being awake but drowsy with periods of inactivity or mild sedation, 3 denoted inactivity but being readily arousable or moderately sedated, and 4 indicated being unresponsive, unconscious or deeply sedated [45,46].

#### 4.5.4. Rota-Rod Test

Automated equipment (Rota-rod, LE 8300, LSI Leica, PanLab Scientific Instrument, Barcelona, Spain) consisting of a rotating roller (7 cm in diameter) was utilized. Prior to testing, rats were trained for three days (day 29 to day 31 of treatment). Each rat was placed on the rotating roller at an acceleration speed of 4 to 20 rpm for a period of 5 min. On the final day of training, the basal values were established. On the test day, each rat was placed on the treadmill with the same acceleration used during training, and the variable evaluated was the latency to fall (time elapsed from placing the rat on the treadmill until it fell). This variable allowed the measurement of the motor coordination level of the rat [45].

### 4.6. Measurement of Behavioral Variables

Digital video cameras (Sony DCR-SR42, 40× optical zoom, Carl Zeiss lens; Sony: Tokyo, Japan; Carl Zeiss: Oberkochen, Germany) were installed above each apparatus (elevated plus maze and locomotor activity) to record activity. Two independent observers measured the behavioral variables using ex profeso software 1.0 version to record the number and time in seconds of each evaluated behavioral variable until >95% interobserver agreement was achieved. After each test session, the apparatus was cleaned with a 15% ethanol solution to remove the scent of the previous rat, which can influence the spontaneous behavior of the subsequent rat. Approximately 2 min elapsed between each test session to allow the odors and cleaning solutions to dissipate.

### 4.7. Statistical Analysis

Data from the elevated plus maze and open field test were analyzed using one-way ANOVA for independent groups, with treatment as the unique factor, or Kruskal–Wallis test if the data did not follow a normal distribution. Rota-rod data were analyzed using two-way ANOVA for repeated measures, considering treatment and condition as factors. ANOVA results with *p* ≤ 0.05 were followed by the Student–Newman–Keuls post hoc test. Values of *p* ≤ 0.05 in the Kruskal–Wallis test were followed by the Dunn post hoc test. These statistical analyses were performed using Sigma Plot 12^®^ software.

Ordinal data obtained from the sedation scale were fitted to a cumulative link mixed model (CLMM), considering treatment, time, and their interaction as fixed effects, and a random intercept per subject. The significance of each effect (treatment, time, and interaction) was evaluated using Type II likelihood-ratio tests, comparing the full model against nested models excluding the term of interest. When *p* < 0.05, a Bonferroni post hoc test was used. This statistical analysis was performed using Jamovi software (version 2.7) with the GAMLj3 module (version 3.7.1). All results are expressed as mean ± standard error.

## 5. Conclusions

The chronic treatment with the flavonoid chrysin exerts anxiolytic-like effects without producing behavioral tolerance, unlike what occurs with diazepam. Further studies are required to identify the precise mechanism involved in the anxiolytic-like effects of chrysin in the long term, to be considered as a potential phytochemical that could be used as a therapeutic alternative for the treatment of anxiety disorders that require a chronic administration. However, specific studies are required to evaluate its safety, pharmacological interaction, possible toxic effects, pharmacokinetic properties, and specific mechanism of action, before starting translational studies and clinical trial for its possible application in humans.

## 6. Limitations

Although this study indirectly evaluated the pharmacological response of the GABA_A_ receptor by administering a sedative dose of diazepam after chronic treatment with chrysin and diazepam, no specific techniques were used to analyze the molecular changes in GABA_A_ receptor subunits after chronic treatment with chrysin or diazepam. In this way, future studies could be focused on studying the expression of different subunits of the GABA_A_ receptor in animals chronically treated with chrysin or diazepam to identify why, if both substances act on the GABA_A_ receptor, the behavioral effects are different.

The importance of including sex as a variable in studies is acknowledged; however, this study used only male rats as an initial approach to determine whether chronic treatment with chrysin could affect GABA_A_ receptor functionality by inducing pharmacological tolerance. Female rats were not included in this initial experiment because the concentrations of ovarian hormones throughout the estrous cycle influence anxiety-related behaviors. Consequently, including females in this initial study would have required increasing the number of animals, a step not justified from a bioethical standpoint for this preliminary exploratory design; nevertheless, future studies should address the chronic effects of chrysin and their relationship to pharmacological tolerance in female rats, taking into account the influence of the estrous cycle phase.

## Figures and Tables

**Table 1:** Treatment effects on the locomotor activity test.

Groups					
Variable	V	C1	C2.5	C5	DZ
Crossing (n)	53.4 ± 3.22	60.7 ± 3.93	65.8 ± 3.75	57.2 ± 4.31	65.5 ± 1.81
Grooming (s)	27.8 ± 6.10	56.0 ± 6.15 *	47.3 ± 8.13	43.9 ± 6.40	32.3 ± 3.65
Rearing (s)	47.3 ± 6.55	49.3 ± 1.44	55.2 ± 2.04 **	44.0 ± 7.13	30.6 ± 4.26

* *p* < 0.05 vs. V; ** *p* < 0.005 vs. DZ. Student–Newman–Keuls post hoc test. V = vehicle, C1 = chrysin 1 mg/kg, C2.5 = chrysin 2.5 mg/kg, C5 = chrysin 5 mg/kg, DZ = diazepam 2 mg/kg. All treatments were intraperitoneally injected. The tests were conducted on day 28 of treatment, one hour after the last injection.

## Data Availability

The data will be made available by the corresponding authors upon request. The data are not publicly available due to the project privacy requirement before closing the general project.

## Abbreviations

The following abbreviations are used in this manuscript:

GABA	Gamma-Aminobutyric Acid
GABA_A_	Gamma-aminobutyric acid type A receptor
V	Vehicle
C1	Chrysin 1 mg/kg
C2.5	Chrysin 2.5 mg/kg
C5	Chrysin 5 mg/kg
DZ	Diazepam
CNS	Central Nervous System
5HT-1A	5-Hydroxytryptamine 1A receptor
5HT-2A	5-Hydroxytryptamine 2A receptor
DMSO	Dimethylsulfoxide
